# Supplementary material for: Identification of photoperiod-induced specific miRNAs in the adrenal glands of Sunite sheep (Ovis aries)
Source: Front Vet Sci. 2022 Jul 22;9:888207. doi: 10.3389/fvets.2022.888207 (PMC9354845; doi:10.3389/fvets.2022.888207)
Supplement: Supplementary file 1 [file Data_Sheet_1.ZIP › Supplementary file/miRDeep2 file.zip!/miRDeep2 file/Novel_Structure/SP42A3/Novel_455.pdf]

Provisional ID	:	NC_019484.2_32191
Score total	:	1.8
Score for star read(s)	:	-1.3
Score for read counts	:	0
Score for mfe	:	1.5
Score for randfold	:	1.6
Score for cons. seed	:	
Total read count	:	16
Mature read count	:	16
Loop read count	:	0
Star read count	:	0

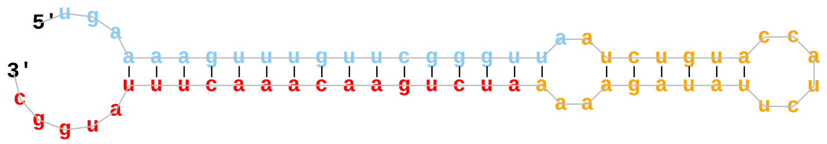

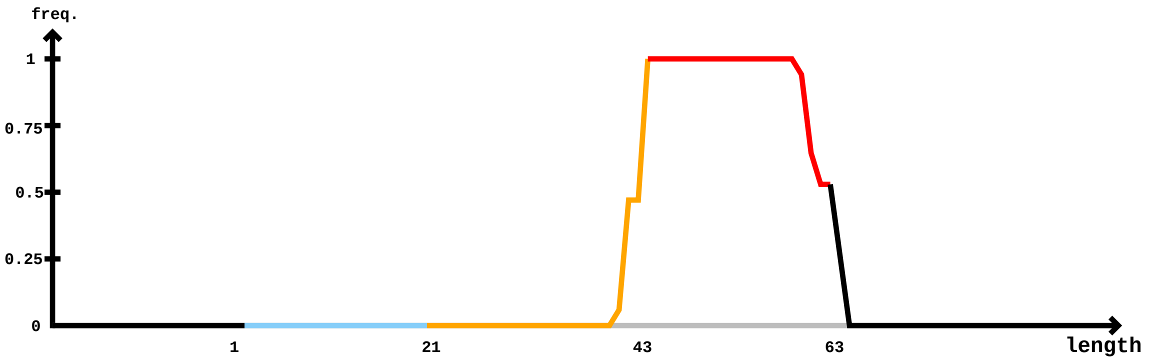

Star Mature

```
5'- acaauccuagguguuggcugaaaaguuuguucggguuaaucuguaccaucuuauagaaaaaucugaacaaacuuuauggcuaacccaguauuuucucuucuuaccugaa   -3'   exp
    .........((.((((((((.((((((((((((((((..((((((......))))))..)))))))))))))))).)))))))))).......................    reads   mm   sample
    ........................................................aaaaucugaacaaacuuuU..................................    1       1    seq
    .........................................................aaaucugaacaaacuuuUu.................................    5       1    seq
    .........................................................aaaucugaacaaacuuuUug................................    2       1    seq
    ............................................................aucugaacaaacuuuUuggc.............................    9       1    seq
```
